# On Playing with Emotion: A Spatial Evolutionary Variation of the Ultimatum Game

**DOI:** 10.3390/e26030204

**Published:** 2024-02-27

**Authors:** D. Y. Charcon, L. H. A. Monteiro

**Affiliations:** 1 Escola de Engenharia, Universidade Presbiteriana Mackenzie, São Paulo 01302-907, SP, Brazil; 2 Escola Politécnica, Universidade de São Paulo, São Paulo 05508-010, SP, Brazil

**Keywords:** emotional expression, evolutionary game, information entropy, population dynamics, social network, spatial game, Ultimatum Game

## Abstract

The Ultimatum Game is a simplistic representation of bargaining processes occurring in social networks. In the standard version of this game, the first player, called the proposer, makes an offer on how to split a certain amount of money. If the second player, called the responder, accepts the offer, the money is divided according to the proposal; if the responder declines the offer, both players receive no money. In this article, an agent-based model is employed to evaluate the performance of five distinct strategies of playing a modified version of this game. A strategy corresponds to instructions on how a player must act as the proposer and as the responder. Here, the strategies are inspired by the following basic emotions: anger, fear, joy, sadness, and surprise. Thus, in the game, each interacting agent is a player endowed with one of these five basic emotions. In the modified version explored in this article, the spatial dimension is taken into account and the survival of the players depends on successful negotiations. Numerical simulations are performed in order to determine which basic emotion dominates the population in terms of prevalence and accumulated money. Information entropy is also computed to assess the time evolution of population diversity and money distribution. From the obtained results, a conjecture on the emergence of the sense of fairness is formulated.

## 1. Introduction

The systematic study of emotion on an evolutionary perspective begins with the book *The Expression of the Emotions in Man and Animals* written by Charles Darwin [1]. In this book, first published in 1872, Darwin discusses the biological aspects and the social value of emotional expression. To Darwin, an emotion reflects an underlying mental state generated by the brain of human beings and other animals, which can be associated with typical facial muscular pattern and body posture [1,2]. Also, to Darwin, emotions have evolved just as anatomical structures have evolved [1,3,4]. Thus, the process of natural selection has shaped not only phenotypic traits, but also psychological responses, in order to enhance the evolutionary fitness of the species. Therefore, our emotional behavior is, at least in part, genetically inherited [1,3,4].

Anger, fear, joy, sadness, and surprise are considered primal emotions [1,2,3,4,5,6], since they were supposedly felt by ancestor species [1,3,4]. Such basic emotions may have increased the chances of survival and reproduction in challenging environments. In fact, these emotions drive vital behaviors: anger destroys obstacles; fear protects from threats; joy facilitates social connections; surprise directs attention; and sadness promotes reflection [1,2,3,4,5,6].

Economic behavior, a particular form of decision-making, is influenced by emotions [7,8,9,10,11]. This statement can be validated by analyzing, for instance, the attitudes of individuals playing the Ultimatum Game. In the original version of this game [12,13,14,15,16], a given amount of money must be divided between two players. The first player, referred to as the proposer, makes an offer on how to split this amount. If the second player, referred to as the responder, accepts the deal, both players divide the money as suggested by the proposer; however, if the responder rejects the deal, neither player receives anything [12,13,14,15,16]. According to classic game theory [17,18], a purely rational responder, seeking to maximize resources for self-interest, should take any offered share, as receiving something is preferable to receiving nothing. As a consequence, a purely rational proposer should offer the minimum possible amount. However, experimental studies revealed that the mutually expected rationality is barely observed, because the average offer is around 40% of the total [13]. Also, around half of the offers below 20% are rejected [14]. Thus, some fifty percent of the responders punish proposals perceived as unfair, despite exiting the game without any gain.

The Ultimatum Game can represent the final stage of a real-world bargaining process, wherein the ultimate offer can either be accepted or rejected [16,17]. During this process, emotions can arise and impact the final proposer’s offer and the final responder’s reply. Functional magnetic resonance imaging has been employed to investigate the neural activity elicited by this game [19,20]. Such neuroimaging studies have shown, for instance, that unfair offers (about 20% or less) increase the activation of brain regions related to cognition (dorsolateral prefrontal cortex), emotion (bilateral anterior insula), and cognitive conflict (anterior cingulate cortex) [19,20]. Thus, unfair offers provoke a struggle in the responder between logic reasons (to take any money) and emotional reasons (to reject low proposals) [19,20]. The interplay between an emotional system and a rational system shapes the responder’s decision [21].

Despite being paradigmatic in studies of cooperation and fairness [12,13,14,15,16,17,18,19,20,21,22], the original version of this game presents two unrealistic features: the resource to be split appears out of nowhere and it disappears in the case of disagreement between the players. In a variation of this game [23], the resource to be divided belongs to the proposer; thus, it returns to the proposer if the negotiation fails. However, in the case of uncompleted negotiation, the survival chances of both players is reduced. On the other hand, a completed negotiation increases the resources of the responder and the survival chances of both. This variation can be suitable to represent a negotiation between employer (the proposer who possesses the money) and employee (the responder who provides essential services to the proposer after receiving the offered amount) [23].

A basic emotion can be simplistically viewed as an information-processing program implemented in the brain to generate specific behaviors in response to specific adaptive problems [1,3,4,24]. Basic emotions modulate the actions in the Ultimatum Game [19,20,25,26,27,28,29,30]. For instance, studies have shown that induced sadness (by previously presenting short movie clips to responders) decreases the acceptance rate of unfair offers [26] and increases the offered share [27]. Joy promotes a more flexible attitude towards making concessions [28]. Fear of failed negotiation increases the offers and decreases the rejection rate [29]. Anger from the responder can be a signal that only elevated amounts will be accepted; anger from the proposer can be a signal of low proposals [30].

Here, five distinct strategies of playing the Ultimatum Game are associated with five basic emotions. This association intends to reflect results found in the literature [13,14,19,20,25,26,27,28,29,30]. The aim is to determine the performance of these strategies. The question to be answered here is the following: which strategies will prevail? In other words, which emotions will have the greatest impact on enhancing survival and reproduction in a competitive environment? This study is computationally performed by considering a spatial evolutionary variation of the Ultimatum Game. The standard version of this game was already investigated from the perspective of evolutionary game theory [31,32,33,34,35,36,37]. In the variation investigated here, a single emotion is assigned to each agent and this emotion reflects a game strategy (as the proposer and as the responder). Also, the money does not vanish after the rejection of an offer, but the survival of the agents depends on closed deals. This variation has not been explored in the literature.

The remainder of this text is organized as follows. In Section 2, the agent-based model used to simulate this evolutionary game is introduced. In Section 3, the metrics based on information entropy used to analyze the population diversity and the money distribution are defined. In Section 4, the results obtained from numerical simulations are presented. In Section 5, these results are discussed from a Darwinian standpoint.

## 2. The Model

Agent-based models have been developed to study complex systems of diverse nature [38,39,40,41,42]. For instance, agents with emotional states have been used to simulate the emotion contagion [43] and the emergence of polarization opinion [44] in online social media and to evaluate a candidate in a selection process [45]. The model proposed in this manuscript is formulated in terms of interacting emotion-endowed agents.

Emotions can suddenly emerge in response to stimuli [1,2,3,4,5,6]. Such responses encompass subjective feelings and physiological reactions [1,2,3,4,5,6]. Different experiences can evoke diverse emotions [1,2,3,4,5,6]; however, here, each agent is considered to have a constant emotional trait. Thus, the emotional state of each agent remains unchanged over time despite its interactions.

**Figure 1:**
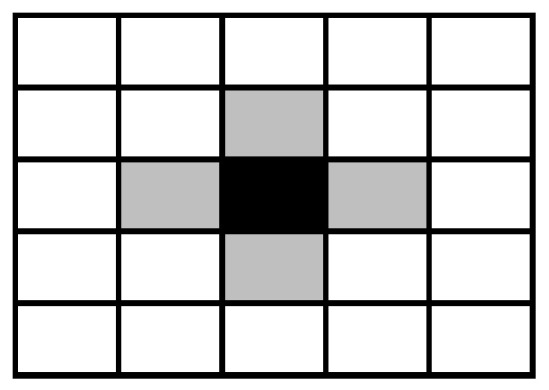
A block 5×5 of a lattice showing the von Neumann neighborhood of unit radius of the black central cell. This neighborhood is formed by the four gray cells. The white cells are not neighbors of the black central cell.

The agents inhabit a two-dimensional lattice represented by an n×n matrix with periodic boundary conditions (that is, the upper and lower edges are joined and the left and right edges are also joined, in order to avoid edge effects). Each one of $N=n^{2}$ agents lives in a cell of this matrix and interacts with the four nearest agents (the north, east, south, and west neighbors), as illustrated by Figure 1. In cellular automata literature, this connectivity pattern is known as the von Neumann neighborhood of unit radius [46] and it has been employed in theoretical investigations on the Ultimatum Game [15,23,32,33,34,36].

Let $d_{i}\left(t\right)$ and $\ell_{i}\left(t\right)$ be, respectively, the amount of money and the number of lives of the *i*-th agent at the time step *t*. At t=0, each agent receives *D* dollars and *L* lives. Then, at each time step *t*, each agent acts as the proposer if it has an accumulated amount of money equal to or exceeding the minimum value *m* (that is, if $d_{i}\left(t\right)\geq m$) and if it is alive (that is, if $\ell_{i}\left(t\right)\geq 1$).

As the proposer, the *i*-th agent at the time step *t* allocates the fraction *f* of its accumulated amount to play the Ultimatum Game with its neighbors. Thus, as the proposer, the *i*-th agent sets aside the amount $fd_{i}\left(t\right)$. Since it has four neighbors, the amount available to play with each neighbor is $fd_{i}\left(t\right)/4$. The effective offered amount depends on its strategy (its emotion), which will affect the responder’s decision. If the deal is closed, both players gain a life each and the amount is split as determined by the proposer. If the deal is not closed, the share $fd_{i}\left(t\right)/4$ stays with the proposer and both players lose a life. Observe that the number of lives *L* represents a predetermined quantity of attempts available to a player to continue playing the game without closing transactions. This limited quantity serves as a buffer against failed transactions and is a parameter that critically influences the player’s survival.

If an agent dies at the time step *t* (that is, if $\ell_{i}\left(t\right)=0$), the predominant emotion in its neighborhood is assigned to the newborn agent that will occupy that empty cell at the time step t+1 (an alternative would be assigning the emotion of the richest neighbor to this newborn agent). If a tie occurs, a random selection is made. This replacement mechanism is called copycat and has been used in other studies on spatial games [47,48,49]. The newborn agent receives *L* lives and inherits the money from the deceased agent. Notice that, at each time step, the *i*-th agent acts as the proposer four times (if $d_{i}\left(t\right)\geq m$, with i=1,2,⋯,N) and as the responder four times. In a simulation with *T* time steps (that is, t=1,2,⋯,T), up to 8NT negotiations can be performed.

A game strategy consists of specifying how to act as the proposer and how to act as the responder. Assume that, as the proposer, the *i*-th agent endowed with the emotion *e* offers the percentage $x_{i,e}$ of $fd_{i}\left(t\right)/4$ to each neighbor; as the responder, it accepts offers equal to or greater than the percentage $y_{j,\epsilon }$ of $fd_{j}\left(t\right)/4$ of the *j*-th neighbor imbued with the emotion ϵ. Therefore, if $x_{i,e}\geq y_{j,\epsilon }$, a negotiation between the *i*-th proposer agent and its *j*-th responder neighbor is carried out; otherwise, it fails.

Consider the basic emotions *e* = {anger, fear, joy, sadness, surprise}. Here, these emotions correspond to game strategies, which are characterized by the values of $x_{e}$ and $y_{e}$. Inspired by results reported in the literature, the following values of $x_{e}$ and $y_{e}$ were chosen:

- Anger: $x_{anger}=20\%$ and $y_{anger}=50\%$ (an angry agent offers little and only accepts high shares [30]);
- Fear: $x_{fear}=50\%$ and $y_{fear}=0$ (a fearful agent makes generous proposals and accepts any offer [29]);
- Joy: $x_{joy}$ is a random number and $y_{joy}=0$. (Here, a joyful agent makes unpredictable offers averaging around 50% and rationally takes any amount. As about 50% of the unfair offers are rejected [14] and sad individuals tend to decline unfair offers [26], it is assumed that the remaining 50% of accepted unfair offers are accepted by non-sad individuals. In fact, joyful negotiators tend to be more cooperative [28]);
- Sadness: $x_{sadness}=50\%$ and $y_{sadness}=20\%$ (a sad agent makes generous proposals [27] and rejects too-low offers [26]);
- Surprise: $x_{surprise}$ is a random number and any offer is either accepted or rejected with a 50%/50% chance (a surprising agent makes unpredictable proposals and the acceptance/rejection is arbitrary and independent of the offered percentage).

Evidently, other values for $x_{e}$ and $y_{e}$ could have been chosen. Notice that anger, fear, and sadness follow deterministic rules; joy and surprise follow probabilistic rules. Notice also that the total number of agents *N* and the total amount of money DN remain constant throughout a numerical simulation (because the number of cells is fixed and the money is only redistributed among the agents as time progresses). Table 1 shows the payoff matrix of this game.

In the next section, two metrics based on information entropy are defined. These metrics are employed to analyze how the basic emotions and the accumulated money vary among the agents.

## 3. Metrics

Let $N_{e}\left(t\right)$ and $M_{e}\left(t\right)$, respectively, be the number of agents expressing the emotion *e* and the total amount of money accumulated by these $N_{e}$ agents at the time step *t*, with e =  {anger, fear, joy, sadness, surprise}. Obviously, $\sum_{e}N_{e}\left(t\right)=N$ and $\sum_{e}M_{e}\left(t\right)=DN$. Also, let $p_{e}\left(t\right)$ and $q_{e}\left(t\right)$ be defined as:

(1) $$\begin{array}{c} p_{e}\left(t\right)=\frac{N_{e}\left(t\right)}{N}, \end{array}$$

(2) $$\begin{array}{c} q_{e}\left(t\right)=\frac{M_{e}\left(t\right)}{DN}. \end{array}$$

Thus, at the time step *t*, $p_{e}\left(t\right)$ is the percentage of agents endowed with the emotion *e* and $q_{e}\left(t\right)$ is the percentage of accumulated money by such agents. Obviously, $\sum_{e}p_{e}\left(t\right)=1$ and $\sum_{e}q_{e}\left(t\right)=1$.

Let $\phi_{e}\left(t\right)$ be the accumulated money per agent with the emotion *e* at the time step *t*. This metric is obtained from:

(3) $$\phi_{e}\left(t\right)=\frac{M_{e}\left(t\right)}{N_{e}\left(t\right)}=\frac{Dq_{e}\left(t\right)}{p_{e}\left(t\right)}.$$

The population diversity at *t* can be assessed by computing the normalized information entropy $H_{p}\left(t\right)$ defined as:

(4) $$H_{p}\left(t\right)=\frac{h_{p}\left(t\right)}{h_{p}^{max}}=\frac{-\sum_{e}\left[p_{e}\left(t\right)\log p_{e}\left(t\right)\right]}{\log5},$$

in which the information entropy $h_{p}\left(t\right)$ is written in terms of $p_{e}\left(t\right)$ [50]. The maximum value $h_{p}^{max}$ occurs when all the emotions appear in equal proportion [50]. Since five distinct emotions are considered, $h_{p}^{max}$ is obtained from $p_{e}=1/5$ for any *e*; hence, $h_{p}^{max}=\log5$.

Similarly, the money distribution at *t* can be evaluated by calculating the normalized information entropy $H_{q}\left(t\right)$ defined as:

(5) $$H_{q}\left(t\right)=\frac{h_{q}\left(t\right)}{h_{q}^{max}}=\frac{-\sum_{e}\left[q_{e}\left(t\right)\log q_{e}\left(t\right)\right]}{\log5},$$

in which the information entropy $h_{q}\left(t\right)$ is expressed as a function of $q_{e}\left(t\right)$ [50]. Since this maximum value $h_{q}^{max}$ occurs for a uniform distribution [50], then $h_{q}^{max}=\log5$.

Here, the normalized inhomogeneity in population diversity $I_{p}$ and the normalized inhomogeneity in money distribution $I_{q}$ are calculated by [51]:

(6) $$\begin{array}{c} I_{p}\left(t\right)=1-H_{p}\left(t\right), \end{array}$$

(7) $$\begin{array}{c} I_{q}\left(t\right)=1-H_{q}\left(t\right). \end{array}$$

Similar metrics based on information entropy have been used to analyze the dynamics of complex systems [52,53,54,55,56,57]. Plots of $I_{p}\left(t\right)$ and $I_{q}\left(t\right)$ reveal how the heterogeneities in agent variability and resource allocation evolve as time passes.

## 4. Numerical Results

Computer simulations were performed in order the examine the influence of *D* (the initial amount of money per agent) and *L* (the initial number of lives of each agent) on the asymptotic values of $p_{e}\left(t\right)$, $q_{e}\left(t\right)$, $\phi_{e}\left(t\right)$, $I_{p}\left(t\right)$, and $I_{q}\left(t\right)$. In addition, the influences of $x_{e}$ (the offered percentage) and $y_{e}$ (the aspired percentage) for anger, fear, and sadness were also investigated.

In the simulations, one of five emotions is randomly assigned to each agent at t=0. Thus, each emotion accounts for approximately 20% of the population at t=0; consequently, $p_{e}\left(0\right)≃1/5$, $H_{p}\left(0\right)≃1$, and $I_{p}\left(0\right)≃0$. In addition, $q_{e}\left(0\right)≃1/5$, because each agent starts with *D* dollars. Hence, $H_{q}\left(0\right)≃1$ and $I_{q}\left(0\right)≃0$. Then, the *N* agents interact with their four neighbors by playing the variation of the Ultimatum Game described in Section 2 for *T* time steps.

**Figure 2:**
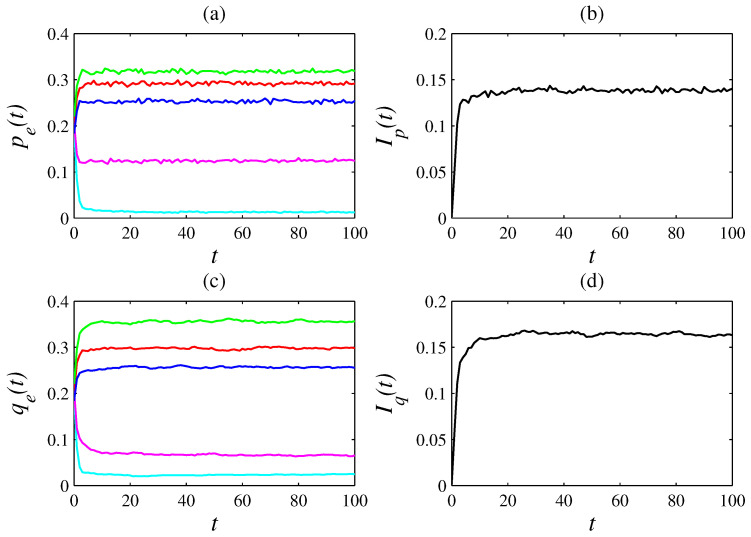
(**a**) Time evolution of $p_{e}\left(t\right)$ for n=50, D=100, L=1, f=1/2, m=1, and T=100 from $p_{e}\left(0\right)≃20\%$ and $q_{e}\left(0\right)≃20\%$ (consequently, $I_{p}\left(0\right)≃0$ and $I_{q}\left(0\right)≃0$). The emotion-endowed agents interact as described in Section 2. In this plot, anger, fear, joy, sadness, and surprise are, respectively, represented by cyan, green, red, blue, and magenta. (**b**) Time evolution of $I_{p}\left(t\right)$. (**c**) Time evolution of $q_{e}\left(t\right)$. (**d**) Time evolution of $I_{q}\left(t\right)$. Notice that these variables converge to nearly constant values.

Simulations show that, as time *t* passes, the variables $p_{e}\left(t\right)$ and $q_{e}\left(t\right)$ tend to fluctuate around constant values. Thus, the dynamics of this game asymptotically tends to a nearly stationary solution. In other words, for t→∞, then, roughly, $p_{e}\left(t\right)\to p_{e}^{*}$, $q_{e}\left(t\right)\to q_{e}^{*}$, $I_{p}\to I_{p}^{*}$, and $I_{q}\left(t\right)\to I_{q}^{*}$, in which $p_{e}^{*}$, $q_{e}^{*}$, $I_{p}^{*}$, and $I_{q}^{*}$ are constants. Figure 2 illustrates a simulation with n=50 (that is, N=2500 agents), D=100, L=1, f=1/2, m=1, and T=100. In this simulation, the emotions anger, fear, joy, sadness, and surprise are, respectively, represented by the colors cyan, green, red, blue, and magenta. Figure 2a shows that, at t=100, $p_{anger}\left(100\right)=0.012$, $p_{fear}\left(100\right)=0.318$, $p_{joy}\left(100\right)=0.292$, $p_{sadness}\left(100\right)=0.254$, and $p_{surprise}\left(100\right)=0.124$; therefore, in Figure 2b, $I_{p}\left(100\right)=0.140$. Figure 2c shows that $q_{anger}\left(100\right)=0.025$, $q_{fear}\left(100\right)=0.357$, $q_{joy}\left(100\right)=0.298$, $q_{sadness}\left(100\right)=0.255$, and $q_{surprise}\left(100\right)=0.065$; hence, in Figure 2d, $I_{q}\left(100\right)=0.163$. In this simulation, $\phi_{anger}\left(100\right)=208$, $\phi_{fear}\left(100\right)=112$, $\phi_{joy}\left(100\right)=102$, $\phi_{sadness}=100$, and $\phi_{surprise}\left(100\right)=52$.

Here, a competitive scenario is specified by a particular combination of *D*, *L*, $x_{e}$, and $y_{e}$ for e= {anger, fear, sadness}, in which the agents compete for monetary resources. As the proposer, the minimum amount to play and the allocated percentage remain fixed in m=1 and f=1/2, respectively. In addition, the number of agents *N* also remains fixed in N=2500. Fifteen scenarios were examined. Each scenario was numerically simulated 10 times. Then, the average values and the standard deviations of $p_{e}\left(t\right)$, $q_{e}\left(t\right)$, $\phi_{e}\left(t\right)$, $I_{p}\left(t\right)$, and $I_{q}\left(t\right)$ at t=100 were calculated (at t=100, these variables already reached their approximate asymptotic values, as shown in Figure 2). These average values are, respectively, denoted by $\langle p_{e}\rangle$, $\langle q_{e}\rangle$, $\langle \phi_{e}\rangle$, $\langle I_{p}\rangle$, and $\langle I_{q}\rangle$. For instance, Table 2 exhibits the results for D=100 and L=1. Table 3 presents the results for n=100 (that is, N=10000). The other parameter values are the same as those shown in Table 2. Notice that the lattice size does not significantly affect the average results; however, the standard deviations usually become smaller when *n* is increased. Evidently, as *n* increases, the computer processing time to finish the simulation also increases.

**Figure 3:**
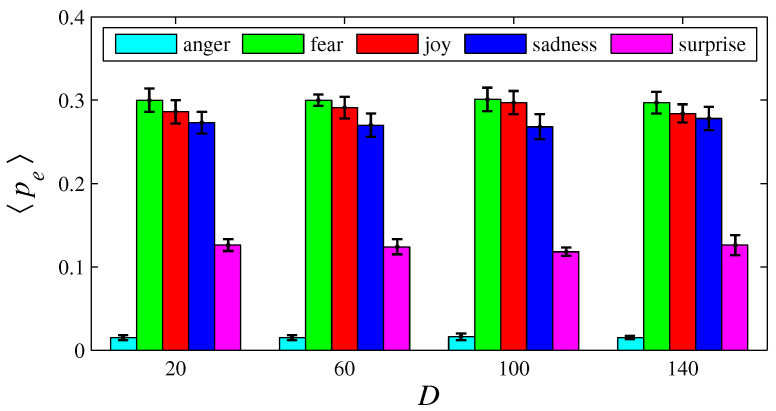
The histograms of $\langle p_{e}\rangle$ in function of *D*, for e = {anger, fear, joy, sadness, surprise}. Here, n=50, L=1, f=1/2, m=1, T=100, and D= {20, 60, 100, 140}. The black error bars represent the standard deviations.

**Figure 4:**
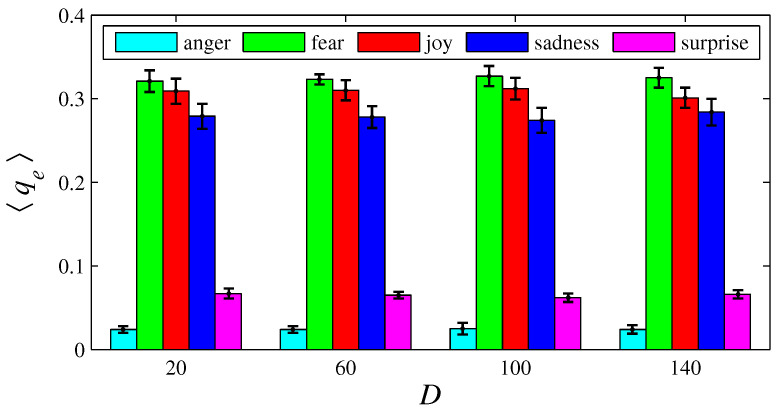
The histograms of $\langle q_{e}\rangle$ in function of *D*, for e = {anger, fear, joy, sadness, surprise}. The parameter values are the same as those used in Figure 3.

**Figure 5:**
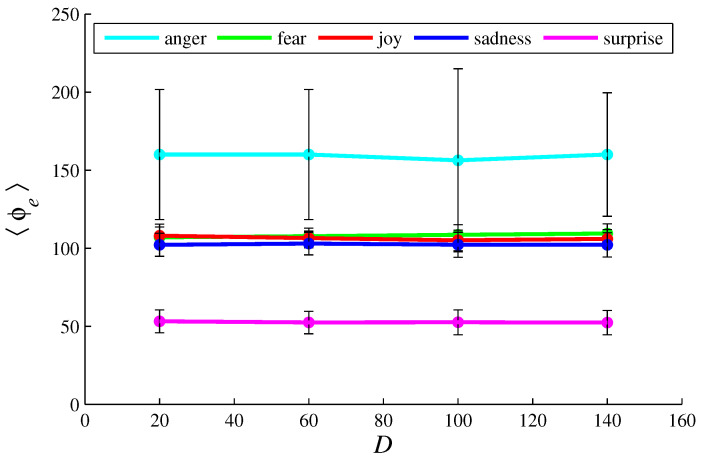
The plots of $\langle \phi_{e}\rangle$ in function of *D*, for e =  {anger, fear, joy, sadness, surprise}. The parameter values are the same as those used in Figure 3.

**Figure 6:**
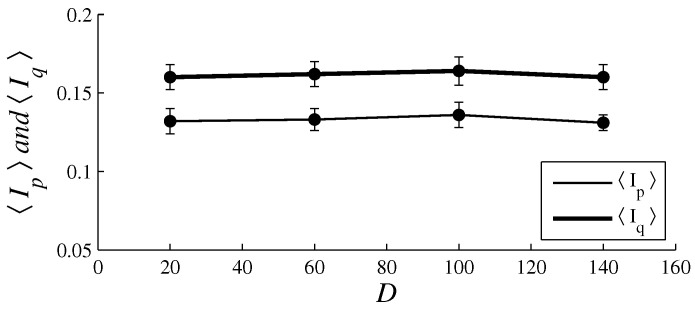
The plots of $\langle I_{p}\rangle$ (thin line) and $\langle I_{q}\rangle$ (thick line) in function of *D*. The parameter values are the same as those used in Figure 3.

**Figure 7:**
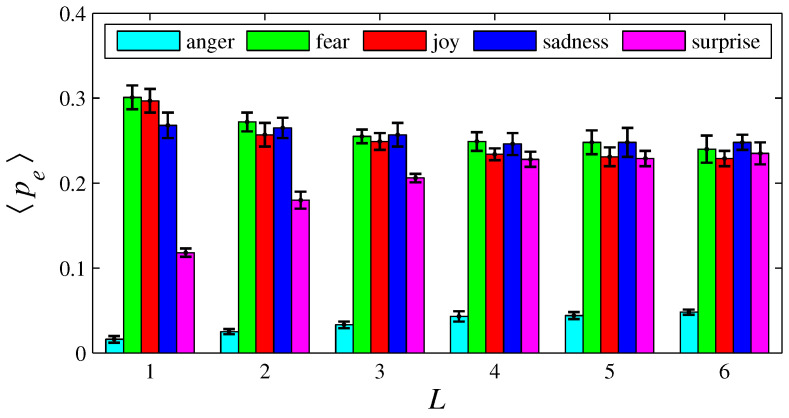
The histograms of $\langle p_{e}\rangle$ in function of *L*, for e =  {anger, fear, joy, sadness, surprise}. Here, n=50, D=100, f=1/2, m=1, T=100, and L= {1, 2, 3, 4, 5, 6}.

**Figure 8:**
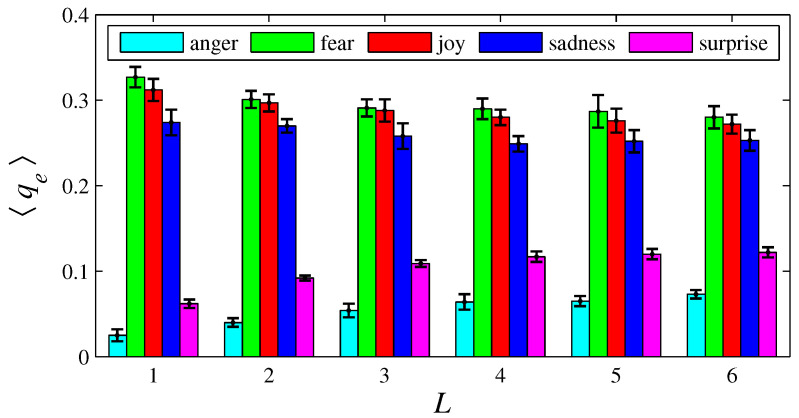
The histograms of $\langle q_{e}\rangle$ in function of *L*, for e =  {anger, fear, joy, sadness, surprise}. The parameter values are the same as those used in Figure 7.

**Figure 9:**
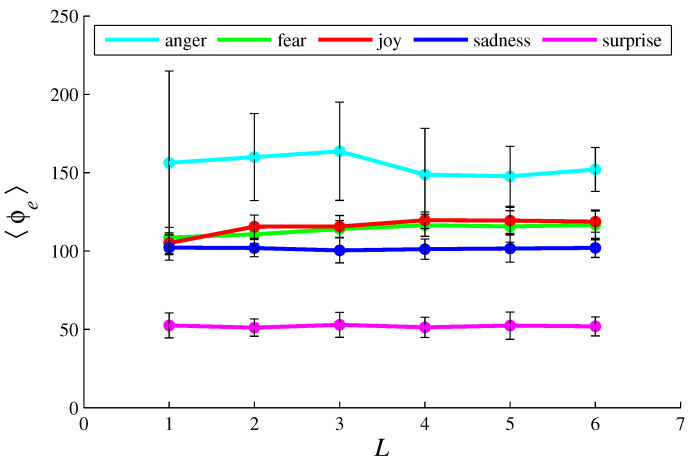
The plots of $\langle \phi_{e}\rangle$ in function of *L*, for e =  {anger, fear, joy, sadness, surprise}. The parameter values are the same as those used in Figure 7.

**Figure 10:**
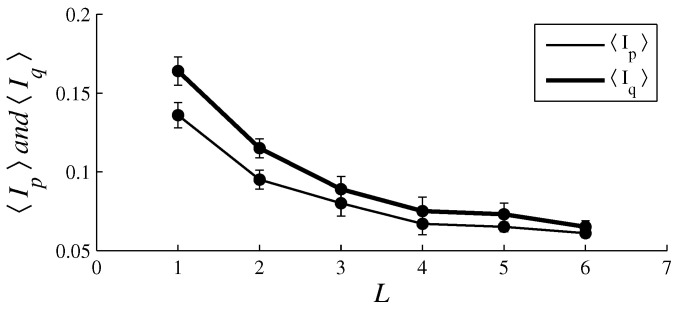
The plots of $\langle I_{p}\rangle$ (thin line) and $\langle I_{q}\rangle$ (thick line) in function of *L*. The parameter values are the same as those used in Figure 7.

Figure 3, Figure 4, Figure 5, Figure 6, Figure 7, Figure 8, Figure 9 and Figure 10 show how $\langle p_{e}\rangle$, $\langle q_{e}\rangle$, $\langle \phi_{e}\rangle$, $\langle I_{p}\rangle$, and $\langle I_{q}\rangle$ vary with *D* and *L*, for L=1, D={20,60,100,140} and for D=100, L={1,2,3,4,5,6}. These figures reveal that fear, joy, and sadness tend to dominate the population in terms of prevalence and wealth. Notice that agents endowed with fear or joy are purely rational responders (because they take any offered share). As the proposer, agents endowed with fear or sadness are generous proposers, because they always offer 50%. Agents endowed with joy or surprise makes offers averaging around 50%; thus, sometimes they propose unfair offers. Angry agents always present unfair proposals.

Figure 3, Figure 4, Figure 5 and Figure 6 show that the parameter *D* has no evident impact on the asymptotic solution of this game. Since the agent’s actions depend only on the offered/aspired percentages of the negotiable amount, the actual value of this amount does not influence their decisions; hence, *D* does not significantly affect the results. However, Figure 7, Figure 8, Figure 9 and Figure 10 indicate that the population inhomogeneity and the economic inhomogeneity are reduced by increasing *L*; that is, $\langle I_{p}\rangle$ and $\langle I_{q}\rangle$ decrease with *L*. Also, increasing *L* benefits anger and surprise, the lowest prevalent emotions for L=1.

Figure 5 and Figure 9 show that, by following the rules presented in Section 2, angry agents accumulate about 150 dollars per agent; fearful agents, joyful agents, and sad agents about 100 dollars per agent; and surprising agents about 50 dollars per agent. These numbers are not strongly influenced by *D* or *L* (or *N*, as shown in Table 2 and Table 3).

**Figure 11:**
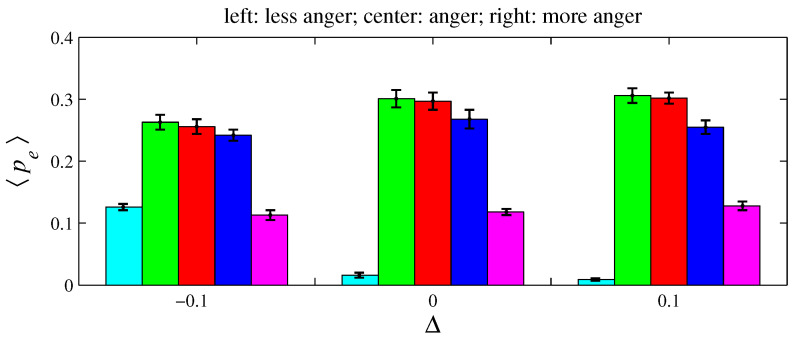
The histograms of $\langle p_{e}\rangle$ in function of Δ. Here, n=50, D=100, L=1, f=1/2, m=1, T=100, and $x_{anger}=0.2-\Delta$ and $y_{anger}=0.5+\Delta$. The other four emotions follow the rules as presented in Section 2. As usual, anger, fear, joy, sadness, and surprise are, respectively, represented by cyan, green, red, blue, and magenta. For Δ=−0.1, $\langle I_{p}\rangle =0.037\pm 0.004$; for Δ=0, $\langle I_{p}\rangle =0.136\pm 0.008$; and for Δ=0.1, $\langle I_{p}\rangle =0.144\pm 0.006$.

**Figure 12:**
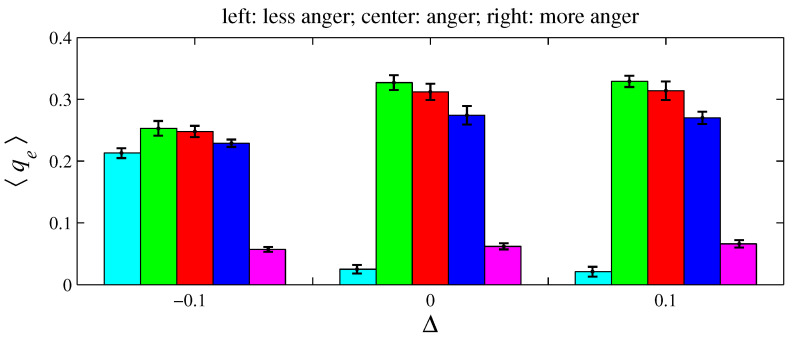
The histograms of $\langle q_{e}\rangle$ in function of Δ. The parameter values are the same as those used in Figure 11. For Δ=−0.1, $\langle I_{q}\rangle =0.054\pm 0.003$; for Δ=0, $\langle I_{q}\rangle =0.164\pm 0.009$; and for Δ=0.1, $\langle I_{q}\rangle =0.167\pm 0.013$.

**Figure 13:**
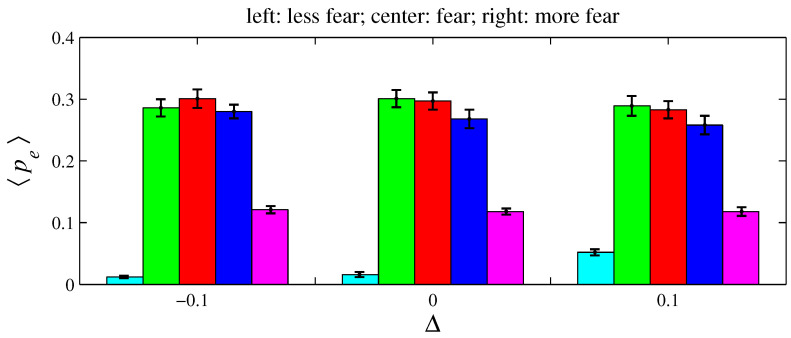
The histograms of $\langle p_{e}\rangle$ in function of Δ. Here, n=50, D=100, L=1, f=1/2, m=1, T=100, and $x_{fear}=0.5+\Delta$ and $y_{fear}=0$. The other four emotions follow the rules as presented in Section 2. For Δ=−0.1, $\langle I_{p}\rangle =0.141\pm 0.006$; for Δ=0, $\langle I_{p}\rangle =0.136\pm 0.008$; and for Δ=0.1, $\langle I_{p}\rangle =0.086\pm 0.004$.

**Figure 14:**
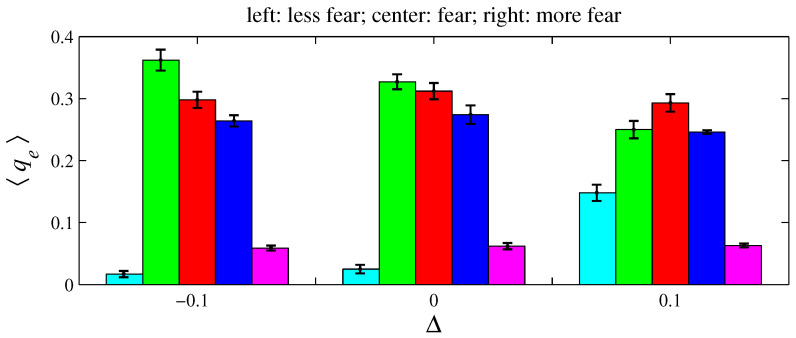
The histograms of $\langle q_{e}\rangle$ in function of Δ. The parameter values are the same as those used in Figure 13. For Δ=−0.1, $\langle I_{q}\rangle =0.183\pm 0.010$; for Δ=0, $\langle I_{q}\rangle =0.164\pm 0.009$; and for Δ=0.1, $\langle I_{q}\rangle =0.064\pm 0.005$.

**Figure 15:**
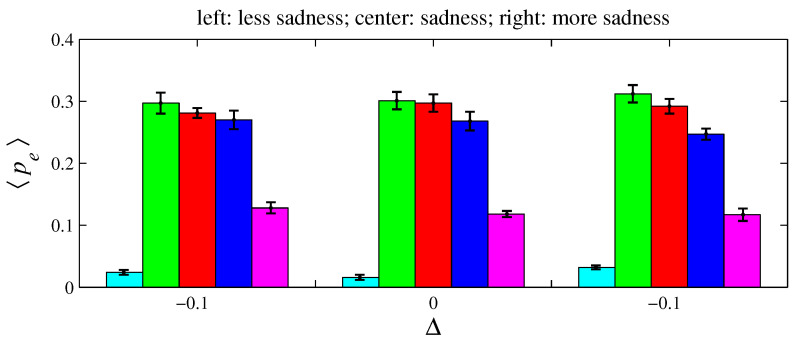
The histograms of $\langle p_{e}\rangle$ in function of Δ. Here, n=50, D=100, L=1, f=1/2, m=1, T=100, and $x_{sadness}=0.5+\Delta$ and $y_{sadness}=0.2+\Delta$. The other four emotions follow the rules as presented in Section 2. For Δ=−0.1, $\langle I_{p}\rangle =0.117\pm 0.007$; for Δ=0, $\langle I_{p}\rangle =0.136\pm 0.008$; and for Δ=0.1, $\langle I_{p}\rangle =0.112\pm 0.008$.

**Figure 16:**
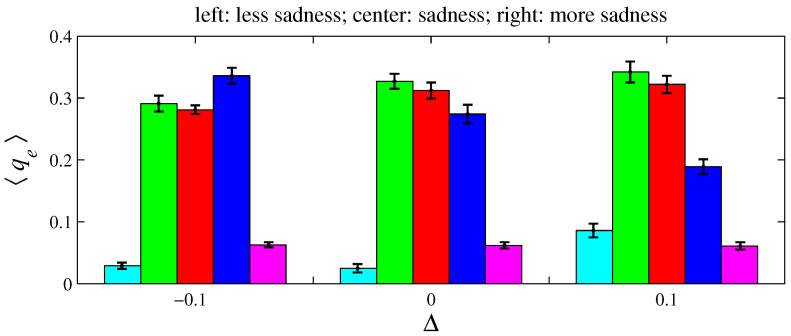
The histograms of $\langle q_{e}\rangle$ in function of Δ. The parameter values are the same as those used in Figure 15. For Δ=−0.1, $\langle I_{q}\rangle =0.156\pm 0.009$; for Δ=0, $\langle I_{q}\rangle =0.164\pm 0.009$; and for Δ=0.1, $\langle I_{q}\rangle =0.114\pm 0.013$.

For e= {anger, fear, sadness}, two variations on $x_{e}$ and $y_{e}$ were simulated for D=100 and L=1. As defined in Section 2, an angry agent is characterized by $x_{anger}=20\%$ and $y_{anger}=50\%$. Therefore, a more angry agent corresponds to $x_{anger}=20\%-\Delta$ with Δ>0 (it decreases its offer) and $y_{anger}=50\%+\Delta$ (it increases its aspired percentage). Conversely, a less angry agent can be described by these same expressions by taking Δ<0. A fearful agent is defined by $x_{fear}=50\%$ and $y_{fear}=0$. Hence, for a more fearful agent, $x_{fear}=50\%+\Delta$ with Δ>0 (it increases its offer); for a less fearful agent, Δ<0. A sad agent is identified by $x_{sadness}=50\%$ and $y_{sadness}=20\%$. For a more sad agent, $x_{sadness}=50\%+\Delta$ with Δ>0 (it increases its offer) and $y_{sadness}=20\%+\Delta$ (it increases its aspired percentage); for a less sad agent, Δ<0. Figure 11, Figure 12, Figure 13, Figure 14, Figure 15 and Figure 16 exhibit $\langle p_{e}\rangle$ and $\langle q_{e}\rangle$ in the function of Δ. Only a single emotion is altered in each of these six figures (the other four emotions behave as presented in Section 2). Figure 11 and Figure 12 are for anger, Figure 13 and Figure 14 for fear, and Figure 15 and Figure 16 for sadness.

Figure 12, Figure 14, and Figure 16, respectively, indicate that playing with less anger, less fear, or less sadness increases the accumulated resources by angry agents, fearful agents, and sad agents, respectively. This conclusion can be drawn by observing the cyan bars in Figure 12, the green bars in Figure 14, and the blue bars in Figure 16. According to Figure 11, the percentage of angry agents decreases with Δ; according to Figure 13 and Figure 15, the percentages of fearful agents and sad agents are not noticeably affected by Δ. In Figure 11 and Figure 12, the average inhomogeneities $\langle I_{p}\rangle$ and $\langle I_{q}\rangle$ increase with Δ; in Figure 13 and Figure 14, these inhomogeneities decrease with Δ; in Figure 15 and Figure 16, these relations are not monotonous.

## 5. Discussion

In human beings, basic emotions are characterized by particular facial expressions [1,2] and distinct patterns of cardiorespiratory activity [58,59] and neural dynamics [60,61], reflecting differentiated brain states and affecting decision-making. In the Ultimatum Game, the proposer’s decision is about resource allocation and the responder’s decision depends on the personal aversion to inequity [12,13,14,15,16,17,18,19,20,21]. Here, a spatial evolutionary variation of this game was played by interacting agents. Their strategies intended to emulate the attitudes associated with five basic emotions. In the proposed model, agents manifesting anger, fear, or sadness were guided by deterministic rules; agents expressing joy or surprise exhibited probabilistic behavior.

In the original version of this game, the players must cooperate to obtain money. In the simulations conducted here, lives are lost if negotiations are not successfully completed. Thus, the agents must cooperate to survive, since lack of cooperation leads to death. Greater cooperation is provided by agents with “high” $x_{e}$ and “low” $y_{e}$; that is, by agents who make generous proposals and even accept unfair offers. Fearful agents fit perfectly into this description. Hence, fear (the green color) has a tendency to prevail in Figure 3 and Figure 7. In contrast, angry agents are characterized by “low” $x_{e}$ and “high” $y_{e}$; hence, anger (the cyan color) is the less prevalent emotion. However, as can be viewed in Figure 5 and Figure 9, angry agents are those that have more money per agent. These two figures also show that surprised agents are the poorest. This result is aligned with the intuitive notion that using a random approach in bargaining processes does not appear to be the most effective strategy for achieving good results. In Figure 11, Figure 12, Figure 13, Figure 14, Figure 15 and Figure 16, the rules guiding the agents imbued with anger, fear, or sadness were modified. These figures suggest that modifications towards rationality increases $p_{e}$ and/or $q_{e}$ of the emotion *e* with altered rules.

Figure 6 indicates that natural selection is barely impacted by varying *D*; however, according to Figure 10, natural selection is clearly affected by varying *L*. In fact, Figure 10 reveals that, by increasing *L*, the inhomogeneities in agent variability and in resource allocation decrease. Increasing *L* implies refraining from immediately punishing non-cooperative agents with death, which enhances the prevalence of angry agents and surprising agents.

Basic emotions have been associated with responses to opportunities and threats related to survival-relevant situations [1,2,3,4,5,6]. Fear is a response to menacing stimuli, which may have been ubiquitous in nature since the very beginning. It is considered to be a (or the most) primitive basic emotion, because it may have emerged at earlier stages in the history of animal evolution [1,3,6,62]. In fact, even invertebrates can feel fear [63]. In this study, the fear of unsuccessful negotiation is equivalent to the fear of dying.

In the model, joyful agents differ from fearful agents by offering an averaging of 50% (instead of the fixed percentage of 50%); sad agents differ from fearful agents by accepting proposals of at least 20% (instead of accepting any share). These slight differences imply similar but distinct performances, as illustrated by Figure 2, Figure 3, Figure 4, Figure 5, Figure 6, Figure 7, Figure 8, Figure 9 and Figure 10. Notice that these three basic emotions are relevant in bargaining processes: fear aims at self-preservation; joy can strengthen social connections by making very generous offers; sadness intensifies aversion to unfairness. On the other hand, expressing anger may not be suitable in negotiations, because it denotes a non-cooperative attitude. Anger, however, can be a driving force for gathering more resources, as indicated by Figure 5 and Figure 9.

Future research projects can consider that each agent can probabilistically exhibit different emotions. Such emotions may be triggered by the neighbors’ behavior or employed as a strategy to improve performance. For instance, a responder can adopt the persona of an angry individual in order to up the proposer’s offer.

Fairness is a moral and social concept. In the context of this study, fairness means equitable distribution of resources. Conjectures on the origins of the sense of fairness based on reputation [31,37], mutation and natural selection [35], food-sharing practices [64], and reciprocity [65] have been proposed. The results found in the computer simulations reported here suggest the following alternative conjecture. In primitive communities, business transactions might have involved the exchange of labor-power for clothes and food. In such ancient times, natural selection may have benefited (fair) individuals who deal with “high” $x_{e}$ (who are generous proposers) and “low” $y_{e}$ (who are rational responders), as fear, joy, and sadness are represented in the model. Thus, the bargaining processes may have been shaped by economic considerations (“low” $y_{e}$) and by affective impulses (“high” $x_{e}$). In this scenario, the selective pressure exerted by *L* (the number of chances of staying alive without completing negotiations) may have been more pronounced than that imposed by *D* (which determines the actual amount to be dealt).

Cooperation means acting together to achieve mutual advantages. Here, cooperation between two fair individuals implies successful negotiation, which contributes to form social bonds and improves the biological fitness of both. Therefore, the development of the sense of fairness may have been supported by basic emotions and it may have been a consequence of creating reliable social connections in order to increase the odds of reproducing and staying alive. 

## Figures and Tables

**Table 1:** In this variation of the Ultimatum Game, the *i*-th agent expressing the emotion *e* (the proposer) makes an offer to the *j*-th agent expressing the emotion ϵ (the responder) at the time step *t* only if $d_{i,e}\left(t\right)\geq m$; that is, if the accumulated resources $d_{i,e}\left(t\right)$ of the *i*-th agent is greater than or equal to the minimum amount *m*. For ϵ = {anger, fear, joy, sadness}, a successful negotiation occurs if $x_{i,e}\geq y_{j,\epsilon }$; otherwise, the negotiation fails. Here, $x_{i,e}$ is the percentage offered by *i*-th agent with the emotion *e* and $y_{j,\epsilon }$ is the percentage aspired by the *j*-th agent with the emotion ϵ. The payoffs and the lives gained or lost by both players are shown in this table. As in the standard payoff matrices employed in game theory, the first number within parentheses corresponds to the payoff/life gained/lost by the *i*-th agent playing as the proposer and the second number to the payoff/life gained/lost by the *j*-th agent playing as the responder. For ϵ = {surprise}, there is a 50% chance of the negotiation being completed.

• for ϵ = {anger, fear, joy, sadness}			
	if $x_{i,e}\geq y_{j,\epsilon }$	if $x_{i,e}<y_{j,\epsilon }$	
if $d_{i,e}\left(t\right)\geq m$	$\left(-fd_{i,e}\left(t\right)x_{e}/4,+fd_{i,e}\left(t\right)x_{e}/4\right)$	(0, 0)	payoff
	(+1,+1)	(−1,−1)	life
• for ϵ = {surprise}			
	50% chance	50% chance	
if $d_{i,e}\left(t\right)\geq m$	$\left(-fd_{i,e}\left(t\right)x_{e}/4,+fd_{i,e}\left(t\right)x_{e}/4\right)$	(0, 0)	payoff
	(+1,+1)	(−1,−1)	life

**Table 2:** The averages $\langle p_{e}\rangle$, $\langle q_{e}\rangle$, $\langle \phi_{e}\rangle$, and the standard deviations obtained in 10 simulations with n=50, D=100, L=1, f=1/2, m=1, and T=100. In this case, $\langle I_{p}\rangle =0.136\pm 0.008$ and $\langle I_{q}\rangle =0.164\pm 0.009$.

Emotion	$\langle p_{e}\rangle$	$\langle q_{e}\rangle$	$\langle \phi_{e}\rangle$
anger	0.016±0.004	0.025±0.007	156±59
fear	0.301±0.014	0.327±0.012	109±6
joy	0.297±0.014	0.312±0.013	105±7
sadness	0.268±0.015	0.274±0.015	102±8
surprise	0.118±0.005	0.062±0.005	53±5

**Table 3:** The averages $\langle p_{e}\rangle$, $\langle q_{e}\rangle$, $\langle \phi_{e}\rangle$, and the standard deviations obtained in 10 simulations with n=100, D=100, L=1, f=1/2, m=1, and T=100. In this case, $\langle I_{p}\rangle =0.131\pm 0.002$ and $\langle I_{q}\rangle =0.158\pm 0.003$.

Emotion	$\langle p_{e}\rangle$	$\langle q_{e}\rangle$	$\langle \phi_{e}\rangle$
anger	0.016±0.002	0.025±0.002	156±23
fear	0.297±0.006	0.319±0.009	107±5
joy	0.290±0.006	0.309±0.010	107±4
sadness	0.273±0.004	0.281±0.007	103±3
surprise	0.124±0.004	0.066±0.003	53±3

## Data Availability

The data used to support the findings of this study are available from the first author (dcharcon@gmail.com) upon request.
